# Mitigation and Remediation Technologies of Waxy Crude Oils’ Deposition within Transportation Pipelines: A Review

**DOI:** 10.3390/polym14163231

**Published:** 2022-08-09

**Authors:** Marwa R. Elkatory, Emad A. Soliman, Ahmed El Nemr, Mohamed A. Hassaan, Safaa Ragab, Mohamed A. El-Nemr, Antonio Pantaleo

**Affiliations:** 1 Polymer Materials Research Department, Advanced Technology and New Materials Research Institute, SRTA-City, Alexandria 21934, Egypt; 2 Marine Pollution Lab, National Institute of Oceanography and Fisheries (NIOF), Alexandria 21556, Egypt; 3 Department of Chemical Engineering, Faculty of Engineering, Minia University, Minia 61519, Egypt; 4 Department of Agriculture and Environmental Sciences, Bari University, 70121 Bari, Italy

**Keywords:** wax deposition, waxy crude oils, remediation technologies

## Abstract

Deposition of wax is considered one of the most significant culprits in transporting petroleum crude oils, particularly at low temperatures. When lowering pressure and temperature during the flow of crude oil, the micelle structure of the crude oil is destabilized, allowing oil viscosity to increase and precipitating paraffin (wax) in the well tubulars and pipeline, which increase the complexity of this culprit. These deposited substances can lead to the plugging of production and flow lines, causing a decline in oil production and, subsequently, bulk economic risks for the oil companies. Hence, various approaches have been commercially employed to prevent or remediate wax deposition. However, further research is still going on to develop more efficient techniques. These techniques can be categorized into chemical, physical, and biological ones and hybridized or combined techniques that apply one or more of these techniques. This review focused on all these technologies and the advantages and disadvantages of these technologies.

## 1. Introduction

The global demand for crude oil has expanded significantly over the last two decades. Concurrently, as conventional light crude oil output declines in response to rising demand, more heavy crude oil reservoirs are being exploited, raising new technological issues at every process level, from extraction to transportation and refinement. In the case of an unanticipated shutdown or restart, the viscosity and high pour points of certain crude oils make them challenging to deal with [1,2,3]. The most critical aspect of flow assurance is the safe and efficient transfer of hydrocarbon crudes from subterranean reservoirs to surface facilities. Because of delayed productivity and the costs associated with remediation, this has been estimated to cost billions of dollars annually [4]. The deposition of wax crystallites within the reservoir (formation damage) and on conduit surfaces are the primary causes of production losses [5]. Common problems caused by wax deposition include reduced production due to decreased flow rates, increased power required to run pumps, and equipment failure, e.g., rod pump and separator malfunctions, choking of flow lines, wells shut in, premature abandonment, and increased manpower attention and workovers [6]. Extracting waxy crude oil from oil fields is time consuming, difficult, and plagued with difficulties. The procedure depends on several elements, including the local environment and the oil composition of each well. The great majority of crude oils and the products that are derived from them both have considerable amounts of waxes that are referred to as paraffin. A variety of hydrocarbons with linear or normal chains, most commonly containing 20 to 40 carbon atoms, as well as alkanes with branched and cyclic chain structures make up paraffin. As the temperature continues to drop, paraffin crystals begin to form, forming a crystalline net that traps the molecules of liquid hydrocarbon until the oil is unable to flow [7].

Flow line pigging is the method that is used most frequently in industrial settings to address the issue of wax deposition. In this method, the scouring action of the pig is used to routinely remove the solid deposit. Wax deposition can be avoided even if flow line pigging is not a viable option for subsea completions by keeping fluid temperatures above the cloud point for the entirety of the flow line [8,9,10]. The use of coiled-tubing technology for well-cleansing techniques has grown in popularity [6]. While the coiled tubing is still in the well, this method permits well production to be moved to fluid collection facilities or flare operations. Using enormous trucks, coils of coiled tubing are lowered into position and high-pressure nozzles are inserted into the well. Solvent-filled tanker trucks supply high-pressure pumps with fluids while cleaning the good tubing as it is lowered into the well in a coiled configuration. Most of the day is spent in operation by a fleet of coiled tubing trucks belonging to integrated manufacturing enterprises in various locations of the world. Chemical inhibitors can regulate wax deposition, but they are not always successful and can be costly; hence, numerous experiments have been undertaken [11] to overcome the deposition problem. Recent nanotechnology advancements in this field may offer a superior option for optimizing output from subterranean reservoirs and ensuring ground flow [9]. All technologies used to prevent or remove wax deposition are listed in Table 1.

**Figure 1:**
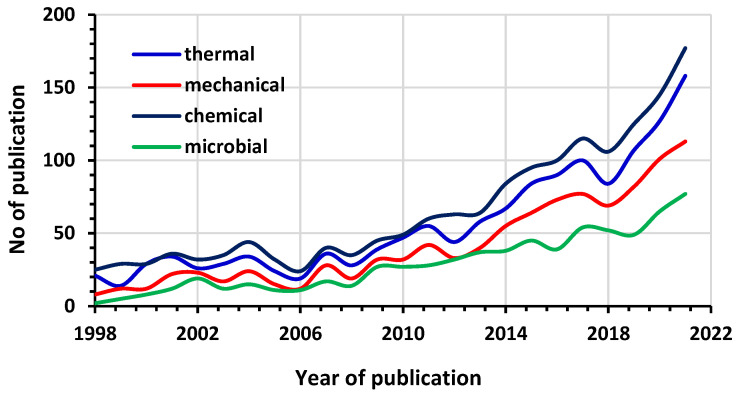
Technologies for inhibition and removal of wax deposition.

On a research scale, the number of published research works dealing with each type of wax deposition treatment, thermal, mechanical, chemical, and biological, is depicted in Figure 1 over a period of time from 1998 to 2021 in order to visualize the upward trend in the scientific community’s interest in the previously mentioned commercially used wax deposition treatments. The studies published in 2022 were not included in these data because this year had not ended yet at the time of preparing. Generally, it was noticeable that there was a gradual increase in the research and development of new approaches for wax deposition. Thus, the highest interest of scientists and researchers was in chemical treatment, followed by thermal.

## 2. Wax Deposition Treatment Technologies 

### 2.1. Prevention and Inhibition Methods

#### 2.1.1. Thermal Methods

##### Insulation

By keeping the fluid at a temperature higher than the cloud point of the crude oil flow line, efficient thermal insulation can prevent any wax from depositing. Although line heaters can be used effectively from the wellhead to other facilities, the physical characteristics of the waxes that crystallize were not altered in any way. One such case involved the use of insulation materials positioned in close proximity to the flow of fluid. Wax deposition can be thwarted by using an insulating a substance that also serves as a coating. Many organic and inorganic materials can be used to insulate a structure from heat. There are a few, however, that are suitable for use in wells because of their mechanical and chemical resistance requirements as well as the difficulty of access, installation, and maintenance. Because the purpose of thermal insulation is to limit heat movement from the inside to the outside, a low thermal conductivity is a prerequisite for any material used in the process. Materials made of plastic are the most appropriate in this situation. In addition to polyurethane and isocyanurate, these materials are frequently employed [12]. Despite this, the chemical industry continues to develop new products that improve efficiency and reduce costs, such as the use of ethylene-tetrafluoroethylene (ETFE) plastic pipe coating [13]. 

##### Active Heating

Using coiled tubing to provide energy by an electrical heater will keep the oil temperature sufficiently high to prevent paraffin crystallization and the subsequent deposition. Furthermore, by heating the crude, the viscosity will decrease, improving the fluid flowability and possibly conducting the heat [14,15]. The method’s technical application is frequently constrained by pipeline dimensions and geometry; however, this is not uncommon. A brief summary is provided in the next section. When it comes to dealing with wax/hydrate issues, two insulated flow lines are the industry standard. Single insulated underground wires (such as Macher tie-backs) are also common. Here, the flow assurance solution includes chemicals. However uncommon, subsea pig launchers can be included in the design for single insulated lines. The employment of heated, dual-flow line configurations is not uncommon. As an example of such a design, look no further than the King field. Heating is given by the circulation of a glycol–water combination. Utilizing direct electrical heating, heat was generated through the steel pipeline wall in the Huldra–Veslefrikk pipeline field (hydrate in condensate production). The electric power cable transmits electricity to the pipeline by piggybacking on a super-martensitic steel (13% Cr, 2% Mo) thermally insulated pipe. The DEH-generated fluid temperature was kept above the equilibrium temperature for hydration formation of the DEH fluid temperature. It was a 10-mile pipeline with an 8 in diameter and an 8-mile length. Age or inadvertent shorts were causing problems with safety and reliability. Long-distance tie-backs should avoid using this technology due to its restricted use [16,17,18]. Active heating’s suitability as a wax/hydrate management method is well established. When it comes to long-distance implementation and expense, this is the issue. These technologies have a solid scientific foundation and broad applicability, but their exorbitant cost makes them unaffordable for most people.

#### 2.1.2. Mechanical

##### Magnetic Application

Treatments by magnetic fluid conditioning (MFC) technology are also relatively new. This technique uses powerful magnets to force the oil into a narrow passageway. Downhole, this helps avoid the build-up of wax deposits and agglomeration by polarizing the wax molecules in a flow-direction orientation. Wax crystals’ agglomeration is disrupted by magnetic field techniques, which modify the kinetics of precipitated wax and make it more difficult for wax to precipitate and develop into larger crystals [19,20,21]. The MFC method had no effect on the WAT, but it did increase the crude oil’s viscosity, according to research. For this procedure, there is still little evidence about its mechanics and a wide range of claims about the method’s success rate [22].

##### Surface Treatment (Internal Coating)

Internal coatings are applied so that wax crystals and other debris do not build up on the pipeline walls. In an experiment that examined the process of paraffin deposition using a model of well tubing, one researcher claimed that paraffin deposits began with the precipitation of paraffin immediately on or next to the pipe wall and grew as a result of the diffusion of paraffin from solution to the area where it was being deposited (simulated well tubing was used in the experiment) [23]. Phenolic, epoxy, phenolic–epoxy, polyurethane, nylon, and Teflon-based coatings are some of the more common options. Chemical properties of the coatings are critical for reducing the wax surface molecular interaction, but physical parameters play a vital role in laboratory and field tests to determine how the coatings affect wax deposition. Other investigations have demonstrated that the insulator character of certain of these coatings prevents deposition better than its chemical composition. Surface finishing to minimize friction between fluid and pipe has failed in every test to prevent deposition, while inner coatings have been found to have no effect on reducing wax deposition [24]. However, investigations and field tests indicate that the physical parameters of the coatings are more important than the coatings’ chemical composition in determining the coatings’ effect on deposition [24,25,26]. Numerous studies [24,25,26] have shown that the insulator character of some of these coatings is significantly more effective at preventing deposition than the chemical nature of the coatings themselves. Smoothing the pipe surface in an effort to lessen the friction that occurs between the fluid and the pipe has failed in every test to prevent deposition, and several investigations have shown that inside coatings have no effect whatsoever on preventing wax deposition [24]. One study [23] concluded that coverings made of nylon appear to reduce the amount of wax deposition that occurs at high flow rates and temperatures that are relatively near to the cloud point temperature. This may be of relevance to cold flow technologies; however, it is necessary to determine whether or not the drop in deposition holds true for slurries with a pour point that is lower than the starting point. There has been no investigation on how wax–hydrate and nylon coatings interact with one another.

#### 2.1.3. Chemical Treatments 

There are a wide variety of chemicals that can be used to improve the flow of pipelines, including wax crystal modifiers, detergents, and dispersants, all of which can be used to address wax deposition difficulties. In this area, you will find substances such as wax inhibitors and pour point depressants (PPD). Crystal modifiers are able to block the creation of big wax molecules in wax inhibitors. They achieve this by interfering with wax crystals and preventing further expansion of the wax. Prior to the wax crystallization process, these polymers are necessary but they are not universally effective; therefore, they must be introduced to the crude oil [1,27]. Some of the inhibitors and flow improvers are injected into waxy crude oil in liquid form, while the rest are delivered as pellets and ‘dropped’ directly into the wellbore [28]. Waxy crude oils‘ low-temperature qualities can be improved commercially by chemical treatments, which was the study’s primary goal. The following paragraph will go into greater detail to highlight that point. Compared to mechanical pigging, chemical treatment is generally more expensive. Only a few chemical inhibitors are capable of completely eliminating deposition. The application of chemical inhibition necessitates the provision of backup pigging capabilities [29]. Wax crystal modifiers, detergents, and dispersants can all be utilized to address wax deposition concerns, as shown previously. Modifiers for wax crystal morphology contain hydrophobic moieties that interact with paraffin molecules and polar moieties that alter and modify the wax crystals’ morphology molecule to molecule [30]. Modifying crystal development and surface features [31] is possible by lowering the pour point and viscosity of the crystal solution. This will result in a decreased propensity for crystals to attach themselves to metal surfaces, such as pipe walls. Wax modifiers, on the other hand, do not prevent precipitation from occurring; rather, they delay the precipitation mechanism from producing irreversible and brittle wax, which makes it possible for the wax aggregates to be removed by the flow of the oil production stream [28,32]. 

##### Wax Inhibitors or Pour Point Depressant 

**Figure 2:**
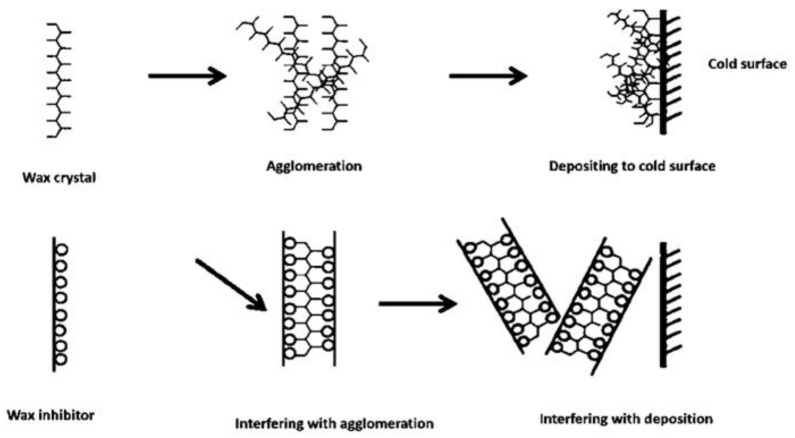
Schematic illustration of the co-crystallization of wax crystal modifier with wax crystals [11].

Pour point depressants are a type of wax crystal modification that includes chemically functionalized substances that are formed from these molecules. They fall under the category of pour point depressants. A wide variety of substances, including highly branched poly-olefins, ethylene vinyl copolymers, alkyl esters of unsaturated carboxylic acid-olefin copolymers, ethylene-vinyl fatty acid ester copolymers, long-chain fatty acid amides, poly *n*-alkyl acrylates, and crystal modifiers, are included in this category. You should aim for the nucleating hydrocarbon agents in order to stop the deposition and accumulation of paraffin crystals. You can achieve this by ensuring that the nucleating agents are kept dissolved in a solvent (Figure 2). 

It is feasible to alter the wax crystallization process in order to lower the pour point, viscosity, and yield stress of crude oil, thereby making it easier to transport [27,33]. This would be accomplished by lowering the yield stress and lowering the viscosity of the crude oil. Flow improvers and pour point depressants share a number of qualities, each of which can have a substantial impact on the effectiveness of the product [34]. Flow improvers and pour point depressants have a number of properties, including (1) the number of pendant alkyl branched chains, as well as the length and distance between them, are the important factors; (2) the solubility of additives (which are generally polymers) in petroleum crude oil; (3) the monomer-to-monomer ratio should be taken into consideration for the additive copolymer; (4) the crystalline and amorphous parts of additive are very important in determining its efficiency; (5) the physical and chemical stabilities of the additive; (6) the prepared polymers; (7) monomer mixtures may vary dependent on the source; and (8) the distributed polymer systems effectively correspond to the polydisperse carbon number distributions in actual paraffin waxy petroleum fluids. 

It was proven that the structure of maleic anhydride polymers can act as wax inhibitors, and this action can have a significant impact on the pour point and viscosity of paraffinic crude oil [34]. The authors demonstrated that the performance of the polymer was improved if its structural components (backbone and pendant groups) were more analogous to those of the wax components. Pour point depressants, also known as PPDs, can be broken down into three categories: ethylene polymers and copolymers, comb polymers, and other branching polymers with long alkyl groups. Although the polymers agglomerate and modify the crystal structure in somewhat different ways, they all accomplish it in a manner that is comparable. These polymers block the wax in slightly different ways. Keep in mind that PPDs are produced in the form of goods that contain active polymers that are encapsulated in a solvent. The amount of polymer that can be dissolved in a solution is influenced by the thermodynamic phase behavior of the solution, which includes factors such as temperature and pressure. On the other hand, the behavior of PPDs when combined with polar crude fractions typically adsorbs on the surfaces of wax crystals, thereby changing crystal–crystal interactions and crystal structure and imparting a reduction in pour points [35].

In most cases, the production of a wax gel is managed with the help of these additives, which are intended to impede and stop the formation of a network between the wax crystals [36]. The crystal–crystal repulsion interactions are frequently of a steric or entropic origin and result from the dynamics of tethered chain sections that extend into the solution close to the solid–liquid interface. When creating strategies for PPD tailoring, polar colloidal content is a consideration that needs to be taken into account. Via addition, polymers can be employed to change the size and morphology of crystals in heterogeneous adsorption, nucleation, or crystal lattice disruption procedures, which result in a decrease in total gel strength [31,33]. Co-crystallization of ethylene-rich sequences is an active sub-mechanism in a number of different forms of PPD. This process can also entail the physical blocking of crystallization sites by non-linear or functionalized entities. PPD-acting copolymers can be divided into two distinct structural categories: (1) linear copolymerized ethylene and (2) comb-structure polymers. Both of these categories are subdivided further into subcategories. Polyethylene that has been copolymerized with a number of monomers, such as butene or vinyl acetate, is widely used as the basis for industrial goods that fall under the category of PPD. Ethylene vinyl acetate copolymers have nucleating properties as well as growth retarding properties [37]. As a result of this, the gel structures become weaker and the crystal sizes become smaller. Comb polymers, on the other hand, make for effective PPD additives due to the fact that they include pendant alkyl groups. Comb polymers often make use of acrylate or methacrylate esters as their backbone moiety. It is possible to make adjustments to the length of the pendant chain as well as the frequency at which it repeats in order to achieve an improved level of functionality in the final result. Commonly employed as a polymeric architecture for the production of efficient PPD products [1,2] are maleic anhydride comb polymers. These polymers have a regular alternating monomer structure on the backbone of the molecule. Multiple studies have concluded that there is a connection between the structure of the polymer and the paraffin-inhibiting action of the material.

##### Types of Pour Point Depressants

Although there is a lot of overlap in terms of the chemistry and mechanisms of these two classes, treating waxy (high pour point) crude oils with these polymeric additives (flow improvers, PPD or crystal modifiers, or wax inhibitors) can lower wax appearance temperature and modify wax crystals [38,39,40]. The most common types of wax inhibitors and PPD can be divided into the following categories. 

##### Ethylene Copolymers

**Figure 3:**
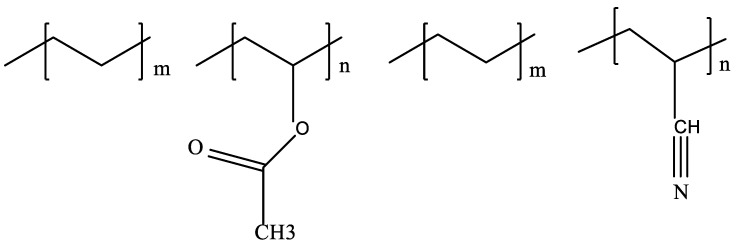
Ethylene/vinyl acetate (left) and ethylene/acrylonitrile copolymers (right) [38].

Among the ethylene/small alkene copolymers, ethylene/vinyl acetate (EVA) copolymers, ethylene/acrylonitrile copolymers, and poly(ethylene-co-butene) polymers [41], poly(ethylene-co-propylene) and poly(ethylene-co-butene) polymers are also included. The random EVA copolymers with a low molecular weight that are shown in Figure 3 are the type of wax inhibitors that are frequently used and researched [38]. The proportion of vinyl acetate in the EVA copolymer determines the efficiency of the copolymer as a wax inhibitor. However, because the polar vinyl acetate moiety promotes solubility and decreases crystallinity, it is required for the WAT depression and reduction, whereas the polyethylene moiety is required for co-crystallization with a structurally closed comparable wax [1,38].

##### Comb-Shaped Polymers

**Figure 4:**
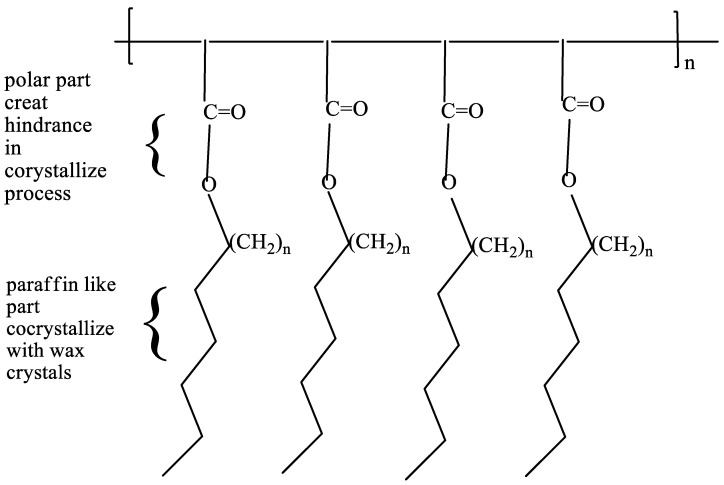
Comb polymer PPD structure with its distinguishing characteristics [34].

Many researchers have investigated wax inhibitors in the form of comb-shaped polymers, as seen in Figure 4 [34,42,43,44]. Maleic anhydride monomers or (meth)acrylic acid monomers are commonly used in the production of these polymers, which generally display stronger wax inhibition than ethylene copolymers [38]. Providing wax crystal nucleation sites on the paraffin-like pendant chains of such polymers while a polar backbone prevents the creation of an interlocking wax network can accomplish this [34]. Polymer comb polymers that have the longest branched chains perform best when applied to crude oil, according to the study. A comb polymer pour point depressant pendant chain length matching crude oil paraffin waxes offered the maximum pour point depression, for example [1,2]. As a result, the authors of [44] found that the optimum wax inhibition was produced utilizing comb structures with long branched chains that interacted with the crude oil fraction most prone to crystallize into a hard paraffin wax phase. Meanwhile, for long-chained waxes, as a result, it is necessary to produce a comb-shaped polymer (or ethylene copolymer) of sufficient length to provide effective inhibition; this necessitates the development of a variety of comb-shaped polymers suitable for various crudes [38].

##### Maleic Anhydride-Based Copolymers 

Maleic anhydride-derived copolymers are another class of middle distillate wax modifiers. Copolymers of maleic anhydride with alkyl methacrylate and alkyl esters [1,2,45], styrene [46,47], vinyl acetate [48,49], and α-olefins [50] were applied for improving diesel fuel flow properties. Maleic anhydride is a very versatile compound, since it can readily react with other compounds and be derivatized in different chemicals with interesting properties. For example, the esterification of polystyrene-maleic anhydride (PSMA) with fatty alcohols can produce derivatives that are oil soluble and can also show surfactant qualities. [51] The presence of the surfactant characteristics, viz. hydrophobic and hydrophilic segments, makes the polymers interesting candidates for pour point depression additives. The copolymers decreased the wax crystal size and prevented the formation of a crystal lattice. Alternating maleic anhydride-vinyl acetate and stearamide derivatives showed good cold flow characteristics as well as high dispersing activities [49].

##### Drag Reducers 

Because the oil temperature at the starting station is higher than the ambient temperature, the temperature of the oil drops throughout the transportation process. When the temperature reaches the point at which crude oil begins to precipitate wax crystals, these crystals begin to grow and eventually deposit on the pipe wall. As a result of increased friction and a decreased effective flow area, sediments on pipelines lower their capacity for transmission. For the development of a pigging program and the safe, affordable transportation of waxy crude oil in a pipeline, an accurate forecast of wax deposition on the pipeline is therefore crucial [52,53]. The petroleum industry has long used drag-reducing chemicals, sometimes known as pipeline flow improvers [52]. The Toms effect, which includes data that reduce drag, was published by Toms [54]. The majority of the early uses of drag reducers involved hydraulic fracturing and were water soluble. In other fields, they were not very common [55]. The year 1979 saw the debut of the first commercial application. This increased the transfer capacity of the pipeline from 1.45 barrels per day to 2.1 barrels per day [56] by reducing resistance on the 1.2 m diameter Trans-Alaska Pipeline by 50%. Drag reducers are effective for reducing the amount of energy needed for pumping since they reduce the perceived viscosity. On the other hand, drag reducing agents (DRAs) prioritize enhancing flow (ORA). The drag reduction rate was typically used to quantify how well crude oil transportation had improved. DRAs are based on a phenomenon known as polymer-induced drag reduction, which was first observed over 50 years ago [54]. At the border layer, these polymers function in a specific way. A modification to a turbulent fluid flow system that reduces the typical rate of frictional energy loss while maintaining turbulent flow is known as induced drag reduction (IDR) [57,58]. DRAs’ mechanism can be broken down into three distinct categories. There are two main types of DRAs. To reduce turbulence in crude oil, the most important parameters are the concentration of the crude oil, pipe size and geometry, molecular weight, chain flexibility, and flow rate. Fibers of the second type intertwine and form three-dimensional structures or networks at modest concentrations in a fluid solution to alter the fluid’s transfer properties and lower flow resistance. A surfactant minimizes eddy currents by interfering with the crude oil and reducing the resistance in the pipeline [58]. The Classification of drag reducer can be found in Table 2.

##### Nano-Heterogeneous 

The term “crude oil” refers to a complex organic mixture that is composed of a variety of hydrocarbon components that can be found in petroleum crudes. These components include saturates (S), aromatics (A), resins (R), and asphaltenes. The chemical composition and structure of these polar (resins and asphaltene) and non-polar (saturates and aromatics) components are intricately connected to the viscosity of the oil, which is the measure of how thick the oil is. Asphaltene is the most polar polycyclic aromatic hydrocarbon (PAH) due to the presence of heteroatoms (N, S, O) and metals (Ni, Fe, V) and the self-association with the development of a viscoelastic nano-aggregate network [67,68]. Its increased viscosity is also a result of the development of a viscoelastic nano-aggregate network. There is a possibility that the viscosity of crude oil will increase if the constituents contain strong C-S and C=S connections [68,69]. According to studies [68,70], asphaltenes and oil viscosity are positively correlated. Experiments utilizing crude oil with and without nanoparticles clearly reveal that the nanoparticles contribute to improve the flow characteristics throughout a wide temperature range by altering the viscoelastic network. These experiments were conducted using both types of nanoparticles. The silica nanoparticles caused a bigger reduction in viscosity than the alumina nanoparticles cause, and they had varying effects on the behavior of the two samples. The precise characterization of crude oils on a molecular level would be helpful in the selection of nanoparticles for better performance. This would make nanoparticles potentially an important agent for flow assurance. The oil industry may use nanotechnology to improve and resolve a number of issues related to flow assurance [8,71] and recovering crude oil from petroleum reservoirs [72]. Nanoparticles have been proven to be able to assist with asphaltenes’ aggregation problems [8]. Numerous problems with oil production have already been successfully resolved by asphaltene adsorption onto nanoparticle surfaces or the reverse [8,73]. Researchers have demonstrated that asphaltenes, the most polar parts of crude oil, are adsorbed onto nanoparticles of various types and natures [8,34,35]. Researchers used silica and aluminum nanoparticles to investigate how to stop asphaltenes from aggregating under various physical and chemical conditions [8,74]. According to Song [74], silica nanoparticles can form on wax crystallization, and rheological behavior on model oils with different contents of asphaltene and resin was investigated. The opposite influence of nanohybrids on the properties of model oils in the absence and presence of asphaltene and resin was observed for the first time. It was demonstrated that SiO_2_ nanoparticles serve as the crystal nucleus to increase the wax crystal number but decrease the wax crystal size so that the WAT rises for the oil without asphaltene and resin, whereas they prevent the aggregation of asphaltene that gives rise to the significant decrease in the wax crystals’ number and the slight increase in the wax crystal size, leading to the improvement of the flowability of oils with asphaltene and resin. Although the effect of SiO_2_ nanohybrids to improve the properties of model oils in our case was not as significant as that for the previously reported PPDs, for the first time, it revealed the mechanism of how nanoparticles work on affecting the properties. We hope researchers can benefit from this work to develop highly efficient PPDs based on nanohybrids. This has established itself as a crucial element for flow improvers. In the current study, two samples of crude oil were evaluated to see how SiO_2_ and Al_2_O_3_ nanoparticles affected their rheological characteristics between 70 and 40 degrees Celsius. FTIR, UV-visible, and fluorescence spectroscopy techniques were used to thoroughly characterize the molecules in each of the two crude oil samples [75]. While "nanoparticle-added" oils revealed changes in flow characteristics, confirming the effectiveness of nanoparticles for flow assurance, nanoparticle-free oils were tested and examined to understand their physicochemical nature after cooling (from 70 °C to 40 °C). The extensive rheological analysis of the two crude oil samples indicates unequivocally that different crude oils may respond differently to the same nanoparticle. According to research, adding nanoparticles to crude oil changes both its composition and rheology in a way that is different from crude oil that does not contain any nanoparticles at all. It was stated that customized nanoparticles (with specific properties) can help effectively tackle the difficulties of stream assurance, even though molecular characterization of raw materials is essential [8]. 

#### 2.1.4. Biological Treatment

Biological methods, such as established systems of paraffin-degrading bacterial consortiums with nutrient supplements and growth enhancers, have been utilized in order to address the issue of the regulation of paraffin deposition in the tube and wellbore region as well as in the surface flow lines [76]. The authors demonstrated that their techniques were quite successful in removing the need for frequent wax scraping over the course of several months. Microbial treatment can be used to limit the wax deposition molecular by-products, which are present in surfactants and wax solvents. The fluid can solubilize the paraffin fractions and remove skin damage caused by paraffin from the wellbore thanks to the bio-production of surfactants and solvents. The API gravity will rise and the cloud point will fall as a result of the cracking of long-chain paraffin [76,77,78]. According to [78], microbial degradation of existing paraffin depositions can disrupt the chemical bonds between carbon atoms and increase the mobility of paraffin. They do not actually sip the oil, though. Another way of breaking down paraffin uses bio-surfactant. Furthermore, due to bacteria’s high mobility in well fluids, this movement enhances the bacteria’s capacity to degrade paraffins. Pseudomonas bacteria’s impact on wax precipitation in waxy crude oils was investigated by Lazar et al. [79] and Etoumi et al. [80]. They came to the conclusion that Pseudomonas species could emulsify hydrocarbons such as crude oil, kerosene, toluene, and xylene [81]. This conclusion was supported by Sifour et al. [82] and other studies. Pseudomonas treatment of crude oil can lower quantities of long-chain hydrocarbons (C_22_+). Etoumi et al. [80] also discovered that the biochemical effect on crude oil is faster within the first 7 days and that Pseudomonas species are capable of reducing paraffin deposition. Additionally, they observed a drop in WAT and viscosity, which is a hint that long-chain alkenes are being changed into short ones [79]. Sood and Lal [83] also investigated how a thermophilic, paraffin-degrading bacteria broke down crude oil. Xiao et al. employed two microbial strains to remove paraffin deposits from stainless steel surfaces [84]. The suspects included Aeruginosa N_2_ and Bacillus licheniformis (KB18). The researchers discovered that a bio-surfactant-producing species can change the wettability of stainless steel to water wet and reduce the aqueous phase’s adhesion to the stainless steel by forming an emulsion. This technique may be essential to prevent the accumulation of paraffin. The results of these investigations are very encouraging in terms of process efficiency. However, bacterial treatment has the potential to eliminate up to 79.0% of the paraffin. Lazar and colleagues identified and chose naturally occurring bacterial consortia and strains from waste hydrocarbon-contaminated locations to remove solid and semi-solid paraffin deposits from flow equipment [77]. Bacterial therapies can be used in a number of real-world circumstances, and field and test-well research is essential to understanding how they function in real-world settings. Despite the lack of success in those circumstances, He et al.’s [81] work deserves attention because they discovered two Bacillus species and a Pseudomonas species in test wells that showed effective paraffin removal capabilities. It was shown that these might increase oil output and eliminate the requirement for more expensive wax removal methods. Furthermore, by injecting a biological solution during a pilot test, Xiao et al. [84] were able to decrease paraffin deposition in oil wells. Many repaired wells were able to operate for 6 to 8 months without any wax accumulation. An average of 4–5 months were seen for surface flow lines after treatment. Santamaria and George had previously looked at the application of commercially available bacteria on five wells with paraffin deposition problems. In five seriously afflicted wells, paraffin-treating bacteria were able to reduce the frequency of significant maintenance production disruptions from once every 2 weeks to once every 6 months. Although no negative impacts or an increase in corrosion issues were observed in the well water, those possibilities could not be completely ruled out. Additionally, the use of those particular bacteria is limited to wells that produce water and have bottom-hole temperatures under 100 °C. 

### 2.2. Removal Methods

#### 2.2.1. Thermal

The use of efficient thermal insulation can fully eliminate the need for wax deposition by maintaining a fluid temperature that is higher than its cloud point throughout the entirety of the flow line. Line heaters can be utilized effectively from the well head to other facilities, despite the fact that the chemical composition of the waxes that crystallize was not altered in any way. The use of hot fluid or electric heating can be helpful in the process of removing wax from down-hole and short-flow lines [85]. The hydrocarbon deposit’s pour point is raised by the thermal energy released by hot oil, water, or steam. It is essential that hydrocarbons are taken out of the wellbore to prevent re-deposition. However, there is a problem with this approach. By concentrating oil and paraffin heavier ends that can no longer be mobilized by the heat available in hot oiling, this can sometimes harm the formation [12]. In some limited applications, electrically heated tube strings have also been used with some success. Paraffin problems have also been managed using exothermic chemical methods in conjunction with inhibitors [13]. Long-term consequences may be worse than short-term benefits when hot oil treatments are used in wells with wax restrictions. As soon as the fluids are placed in storage, wax crystals can form due to the temperature and fluid flow conditions, leading to gels and sludge [86].

#### 2.2.2. Mechanical

During cleaning in a flow line that is not entirely clogged, this procedure is available. Perforation clogging and a rise in oil-in-water emulsion stability were some of the drawbacks associated with these approaches Pigging is used to mechanically scrape away the paraffin wax from the inside of the flow line. Pigging has a variable level of operational efficiency depending on the pigs’ design and other factors. Pigging in subsea systems and the pigging requirements for platforms, flow lines, and FPSO designs were all reported by Zyrin [87]. It is necessary to build a dual-flow line system with a pigging-compatible architecture. Pigging must be performed on a frequent basis to avoid the buildup of a lot of wax. Pigs cannot be forced to pass through a heavy coating of paraffinic wax because the wax collects in front of them and prevents the pig from being pushed. The submarine petroleum oil system must be shut down prior to pigging, stabilized using a methanol injection and blow down, and then resumed after pigging is finished. You can lose 1 to 3 days of output during this process. Pigging is more laborious and less efficient if the deposit is thick and hard [88].

##### Coiled Tubing

**Figure 5:**
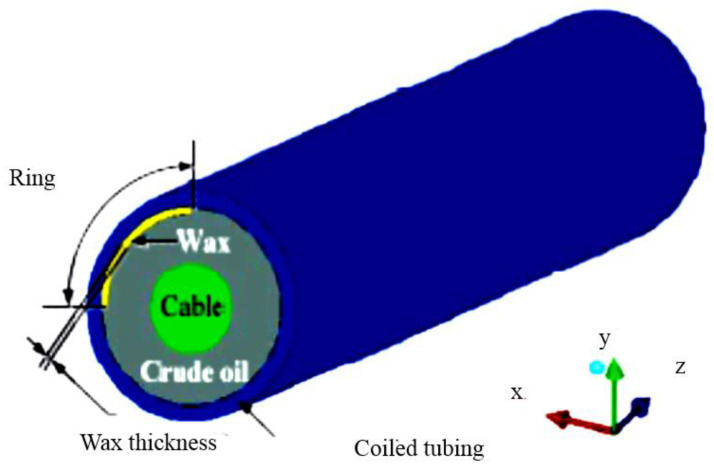
Cross section of flow stream line [14].

An electric submersible pump is attached to the end of coiled tubing as a potential method of recovery. There are steel strings, heat-resistant rubber, and copper conductors in the electric cable to the pump. Since its cross section is higher than the tubing’s inner section, its influence on the oil flow must be clearly understood [14,15]. When it comes to flow assurance issues, the use of coiled tubing is increasing. The method’s technical applicability is frequently restricted by pipeline dimensions and design, which covers coiled tubing and pigging, allowing an electric power cable to be installed [89]. The fluid temperature produced by DEH was kept higher than the temperature at which hydration forms at equilibrium. The temperature of the DEH fluid was caused by an 8-mile long, 8-mile diameter pipeline. Safety and dependability issues were being brought on by old age or unintentional shorts. This technique has not been widely used, and long distance tie-backs are not recommended [14]. The wax is broken up into zones at its two ends, and the maximum velocity is visible in the space between the wax and the tube. Wax buildup causes a greater pressure loss. These wax effects become more pronounced as the wax section or thickness increases (Figure 5). In order to deal with wax accumulation, it is suggested that a wax layer of the same thickness be used. Friction pressure loss can be calculated using a concentric liner calculation model, which can be applied in engineering with ease [90].

##### Pigging

**Figure 6:**
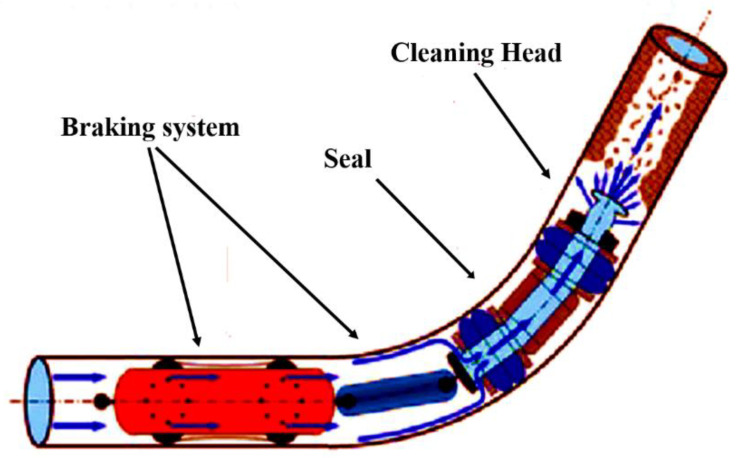
Pigging process [91].

This procedure is only suitable for clearing flow lines that are not entirely clogged. Pigging is used to mechanically scrape the wax from the flow line. Depending on the pigs’ design and other pigging criteria, the efficiency of the pigging operation may vary significantly. Figure 6 [87] shows how Gomes and his coworkers looked into submarine pigging strategies as well as their requirements for platforms, flow lines, subsea equipment, and FPSO designs [87]. Wax needs to be physically removed by brushing the flow line. A dual-flow line system that allows pigging must be constructed in order to make pigging easier. The primary elements that influence cleaning tool design are relatively fundamental and typically center on the following:

- The size of pipe;
- The pipeline section length;
- The smallest bend radius used when building the line;
- The item travelling through the pipeline;
- How many I/D modifications resulting from changes in wall thickness are currently planned?
- Types of valve;
- Is the pipeline cross-country or subsea?
- Pig trap design;
- What debris is to be removed from the pipeline and pipe wall by the cleaning device?

Pigging must be accomplished on a regular basis in order to prevent the accumulation of large amounts of wax [89]. Wax buildup in front of the pig will reduce the pressure needed to move it forward if it becomes too thick. After the pigging is completed, the subsea oil system is shut down, stabilized with methanol injection, and then restarted. One to three days of productivity may be lost as a result of this entire process. Using the fluids’ analysis and flow assurance calculations to generate deposition models, pigging intervals can be set such that the pig does not get stuck in the flow line too frequently or too infrequently [90]. The wax "slug" is pushed forward of the pig, as modelled by OLGA [91,92,93]. Practical analysis of the nitrogen gas effect on stripping process of the H_2_S during the pigging operation of a long crude oil pipeline was reported [94,95].

##### Jet Cutting 

A ‘jet’ is a stream of fluid flowing faster than the surrounding fluid; when it comes into contact with a solid boundary, the frictional resistance it encounters accelerates its decay. There is a simple power law expression for the change in the velocity of the wall jet at a distance x from its origin, according to Glauert [96]. As a result of this, the shear stress on the pipe wall and the removal rate of wax deposits can be determined by this change in velocity at incremental distances from the jet origin. From the fact that critical velocity has been established in many pipeline operations that determine movement or settlement of solid phases, the notion of using a wall jet to remove wax deposits was conceived. Wax deposits were cut off before they had a chance to harden, according to studies of deposition [96]. This finding is taken to suggest that there is a flow velocity at which the removal rate of wax owing to fluid shear surpasses the removal rate of the wax settlement. It is preferable to investigate the removal mechanism in isolation, even if settlement and removal processes share the same physical parameters. In this manner, a wax removal model that may be used as a corrective technique can be developed. Other industries employ similar shearing methods. Flow velocities at various places in a sewage system [97] are used in the water business to estimate the settlement, removal, and transport of solids (slime). Sewage systems are self-cleaning because engineers can design them to be periodically self-cleaning through the removal of debris and slime that settles in the system, such as during heavy rains. A distinction must be made between this thesis’s procedure and the high-pressure jet cutting processes often employed in machining. These high-pressure, high-velocity jets can reach pressures of up to 1000 bar through fine bore nozzles, [98] often at supersonic speeds. It would be impossible to achieve such a pressure differential over a pipeline pig, and the fine bore nozzles would be prone to blockage due to the foreign debris found in crude oil. An annular jet that bypasses the pig was used in this work to produce enough shear stress to remove soft wax deposits. Glauert [96] provided theoretical and experimental explanations for the behavior of such a jet. These theoretical and experimental experiments had excellent agreement and they gave a helpful platform for further investigation. 

#### 2.2.3. Chemical

Wax deposition can be removed with the help of solvents and dispersants, two different types of chemicals. Dispersants, on one hand, are employed to break up wax particles so that the production flow may carry them [1,2]. The earliest known way of removing wax from wells is to use a chemical solvent such as gasoline, kerosene, or benzol. Gasoline outperforms kerosene as a solvent for removing wax buildup. The cost of the solvent has a bearing on its usefulness for wax removal using solvents [99,100,101,102]. Because the solvent can also be recovered for use as crude oil, the additional value should be removed from the total cost of purchasing the solvent. This method cannot be used with naturally flowing wells since it is difficult to pour liquid solvents into them; however, it can be used with wells that require pumping. In order to establish which solvent was the most effective at removing paraffinic deposits, Straub and his colleagues conducted a series of tests using a variety of solvents, such as xylene, kerosene, diesel condensate oil, toluene gasoline, and mixes of these products. In 82% of the tests, it was discovered that the paraffin was dissolved by xylene or xylene mixtures more quickly than by any other solvent. They came to the conclusion that the temperature, paraffin characteristics, and type of solvent all had a significant impact on the solvent reaction. Both carbon tetrachloride and carbon disulfide have been utilized as universal solvents, as stated by Al-Yaari and Fahd [11]. In order to dissolve the low asphaltene concentration that is present in paraffin deposits, solvents such as kerosene, condensate, and diesel oil are utilized [103]. Wax dispersants and flow improvers with a nitrogen-based, one-component polymeric structure have been produced. Dispersal is influenced by the polar effect of nitrogen/oxygen, according to the researchers. The permeability of the paraffin wax crystals during their creation affects their efficacy as well [1,2].

##### Solvent 

Solving wax and asphaltene deposits is frequently the most effective way of cleanup. In order to increase the dissolving capacity of the solvent system, solvents such as xylene, toluene, or naphthalene can be used alone or in combination with other solvents. The solvents are often highly combustible, incompatible with demulsifying chemicals, and do not alter the surface oil wettability, all of which work together to prevent re-deposition of oil [102]; they are highly expensive. As a result, solvent remediation techniques are typically used in situations when thermal treatments are ineffective. A green solvent, terpene, derived from natural and renewable sources has been utilized as an alternative to the conventional solvents for wax remediation. Terpene is composed of repeating five-carbon isoprene units, grouped as unsaturated aliphatic cyclic hydrocarbon, abundant in renewable plant resources such as oleoresins from pine plant (alpha and beta pinene) and orange peels (d-limonene) while others include turpentine, citronella, carotene, and many more. Terpene has low toxicity, is less flammable, is rapidly biodegradable, and has high solvency for an organic deposit comparable to aromatic solvents. Terpene is a good surfactant that is environmentally friendly. Terpene can be blended with other co-solvents [102]. Comparatively, terpene possesses good solvency and is biodegradable, less toxic, and less flammable. 

##### Dispersants

**Figure 7:**
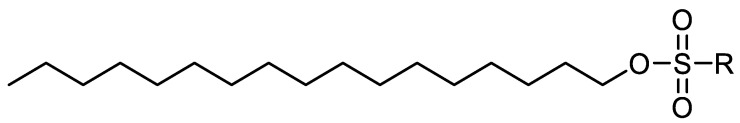
Alkyl sulphonate is an example of a wax dispersant; the *R* group is typically an aryl or an alkyl group.

There are three types of surfactants: cationic, anionic, and non-ionic. Cationic surfactants are often referred to as wetting agents. Wax dispersants, despite having a chemical structure that is comparable to that of PPD (Figure 7) [101], have a bigger polar functional group, which is what determines the surfactant nature of wax dispersants. The adhesion of waxes to pipe surfaces is reduced by the presence of these surfactants, which also cling to pipe surfaces. They might accomplish this by making the pipe surface wet with water, by forming a thin layer from which wax crystals can be easily sheared off, by adhering to the wax crystals and reducing their tendency to stick together, or by doing some combination of these things [1,2]. Alternatively, they might just use a combination of these methods. It is the surfactants, not the wax itself, that are responsible for dispersing the wax in the oil or water; dispersants are also used. It is possible to eliminate wax deposits by utilizing the dispersants in conjunction with the modifiers. Because they scatter it into smaller parts, low shear circumstances make it easier for the crude oil to join with the modifier polymer. This is because low shear conditions make it easier for the crude oil to blend. Some researchers have worked on developing their own dispersant mixtures [103]. For example, they developed an inhibitor that interferes with the mechanism of wax crystal growth by preventing the formation of a three-dimensional network. This results in a reduction in the pour point of crude oils and an improvement in their flow properties. However, it comes at a high cost and poses a significant risk [1,2,75]. When heat and a surfactant are used together, a bipolar interaction occurs between the surfactant and the wax at the boundary between the two phases (water and wax). This interaction allows for the suspension of particles. One of the advantages of utilizing this method is that water normally arrives at the site of a deposition at a greater temperature. Water also has a higher specific heat than oil due to the fact that oil is denser than water. Polyamides, alkyl phenyl derivatives, and sulfonates are the three types of chemicals that are used to make dispersants. 

#### 2.2.4. Hybrid Treatment

##### Mechano-Chemical Treatments

Wax deposition can be controlled with chemical inhibitors, although these compounds can be ineffective and expensive. Wax deposition is controlled for subsea completions when flow line pigging is impossible by keeping fluid temperatures above the cloud point across the entire flow line. The adoption of coiled-tubing technology for well cleanups today has many advantages. This technology has the advantage of rerouting well production to fluid collecting or flaring activities while coiled tubing is in situ. The technique involves diverting well fluids away from the well and inserting high-pressure nozzles on coiled tubing into the well. High-pressure pumps can clean the well tubing by passing through fluids that are pumped through tanker trucks filled with a solvent. Many integrated manufacturing organizations have a fleet of coiled tubing vehicles that are always in use, highlighting the relevance of this approach in many parts of the world. Coiled tubing, which is utilized in wax remediation, is an effective mechanical therapy. Both flow line insulation and the injection of chemicals for wax dispersion can reduce deposition rates by up to five times, respectively. It is important to remember that these chemicals do not stop the wax from forming completely. As a result, scouring the flow line is still required to remove the wax.

##### Thermo-Chemical Treatment

**Figure 8:**
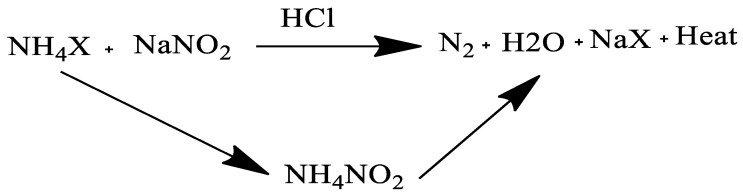
Wax deposition removal using a thermos-chemical process.

The nitrogen-generating system (NGS), a thermos-chemical cleaning method, was created by Petrobras in 1992. By carefully controlling the production of nitrogen gas, the NGS technology combines chemical, thermal, and mechanical elements to produce the reversible fluidity of paraffinic wax deposits. The exothermic, effervescent reaction that occurs when two chemicals are mixed together removes and eliminates deposits. The acid-catalyzed breakdown of ammonium nitrate is a frequent reaction. Mixing ammonium chloride and ammonium nitrate in water is the first step in the process, as illustrated in the reaction equation in Figure 8. The pH is maintained between 5.0 and 8.0 to control the reaction. It is critical, however, to maintain the reaction under control, as the reaction might pose a serious threat to pipes and materials or even the pipeline system in extreme cases. Nitrogen gas and heat are produced by mixing two nitrogen salt-containing aqueous solutions in the pipeline’s impacted area. Aside from common salt and pure water, Petrobras’ promotional materials suggest that the reaction is environmentally beneficial (i.e., brine). As with other chemical procedures, this one also relies on the use of chemicals, melting deposits as a result of an exothermic chemical reaction of this magnitude [104,105,106,107,108]. It is extremely difficult to induce an exothermic chemical reaction at the appropriate spot in order to melt the wax within the tube and remove the wax deposition using chemicals; hence, it is highly unlikely that wax deposition can be removed using chemical methods. As a result, if this reaction occurs near the bottom of the well, the heat is likely to evaporate before accumulating the paraffin in the well. Because of the high cost of chemicals required to ensure the melting process, this technology is not economically viable. A delayed reaction chemical compound based on the reactions of NH_4_Cl and NaNO_2_ was tested by Ashton and colleagues [107]. Wellbore and low-permeability formation parts could be warmed using this technique. It was a success. It was determined by the volume of chemicals poured into the heating zone. Despite the functional advances, the method’s practical application and cost effectiveness remain an impediment to its widespread implementation. The use of this technology in Indian oilfields, however, has been documented by Tiwari [108]. According to reports, a successful trial after laboratory testing used the following exothermic reaction: NaNO_2_, ammonium chloride, and sodium chloride (NaCl) were all used in the exothermic reaction. Exothermal reactions can be combined with other thermos-chemical effects to create more powerful dewaxing thermo-chemical packages. To give one example, Petrobras pioneered the nitrogen generation system in 1992 [4,108]. Additionally, the effervescent reaction of the ammonium chloride/sodium nitrite solution helps remove wax deposits in addition to the exothermic impact. Even though it was in use for a long period, this technique also showed its fault of providing a solution that is quite confined in space, up until the point when the chemicals are injected. Taking this into consideration, Halliburton created the SureTherm in 2012, a package with a delayed impact. West African trials have allegedly gone well for the product.

Any of the methods can be employed, after careful consideration of the operating conditions of the well or field, to achieve efficient results. Currently, the technical solutions with the most widespread use for removing wax deposits in transporting pipelines are the thermal and mechanical removal techniques; despite this, they are now the most popular technological method for removing wax deposits from transporting pipelines. The pigging is broadly well known to remove paraffin waxes. Electric heating has several advantages, including low energy consumption, convenient laying, minor environmental impacts on the seabed, and the strong ability to maintain high temperatures (despite the possible cable connection failure). It has become an industry-approved process as the primary way heat loss in submarine pipelines can be minimized. Heat loss in long-distance transportation will be solved through in-depth research on methods to supplement heat loss and pipeline insulation capacity in the future. In addition, based on the flow properties and flow patterns, established models may be unable to accurately predict the wax deposition in the multiphase flow of crude oil. This is complicated by other materials contained in crude oil (such as solid gravel, asphalt, and hydrate). For internal coatings, investigations and field tests indicate that the physical parameters of the coatings are more critical than the coatings’ chemical composition in determining the coatings’ effect on deposition. Numerous studies [24,25,26] have shown that the insulator character of some of these coatings is significantly more effective at preventing deposition than the chemical nature of the coatings themselves. For chemical treatment, the improvement of mining requirements and the strengthening of environmental protection, methods for optimizing the flow of submarine crude oil pipelines, are constantly evolving. Compared with the process of adding light crude oil, the technique of adding DRA, PPD, and nanohybrid offers tremendous advantages and confirms the effectiveness in flow characteristics.

In most cases, two or more techniques are combined, e.g., chemical with heating was used to achieve incredible treatment and to reduce cost. Similarly, heating with the pigging method was tested to reduce energy consumption and environmental pollution. Methods that have been tried and proven for decades are listed in Table 1. Although most researchers focus on chemical technology and liberating research (Figure 1), chemical treatment is the preferred prevention technique, especially for uninterrupted production, long-distance pipeline/flow lines, and reducing restart pressure and yield stress. Chemicals usually offer an improved rheology property by disrupting the orderly aggregation of the growing crystals, which decreases the viscosity, pour point, and the WAT. Unfortunately, the application of chemicals is usually limited to a specific oil well and deposit due to the different properties of the crude oils and their origin. This implies that in all the types of crude oil, no single inhibitor was developed to be equally suitable for each oil. According to the investigations on wax deposits, all circumstances were not treated in the same way. Considering conventional techniques and the most recent state-of-the-art technologies, it is reasonable to anticipate that the remaining technologies, with the extension of the oil production pipeline, will increase production depth. This will likely increase and may become the mainstream method for the flow of crude oil in submarine pipelines. With sustainability in view, environmentally friendly techniques will be preferred, such as the creation of new chemical inhibitors. The green solvent terpene is an excellent example of non-toxic and clean methods; avant-garde heating apparatuses and microbial treatment have effectively prevented wax deposition. The method is environmentally friendly [102]. It can be injected into wells and will experience significant developments and practical applications in the future. As a final remark, it is important to stress that any sensible evolution within the prevention and removal techniques will require advances in wax deposition modeling to enhance its adequacy and efficiency to decide which approach should be used to prevent or remediate the wax deposition.

## 3. Conclusions

Most researchers focus on chemical technology and liberating research (Figure 1). Mechanical removal, in spite of this, is now the most popular technological method for removing wax deposits from vertical oil wells. Methods that have been tried and proven for decades are listed in Table 1. All circumstances were not treated in the same way, according to the investigations on wax deposits. When taking into account conventional techniques and the most recent state-of-the-art technologies, it is reasonable to anticipate that the remaining technologies, such as the creation of fresh chemical inhibitors, avant-garde heating apparatuses, and efficient bacterial treatments that can be injected into wells, will experience significant developments and practical applications in the future.

## Figures and Tables

**Table 1:** Technologies for inhibition and removal of wax deposition, modified from [7].

Categories for Wax Deposition Treatments		
**(A) Inhibition (Prevention of Wax Crystal Precipitation or Deposition)**		
	Thermal	Insulation Active heating
	Mechanical	Magnetic application Surface treatment (internal coating)
	Chemical	Wax inhibitors/Pour point depressant Drag reducer Nano-heterogeneous
	Biological	Naturally occurring microorganisms
**(B) Removal (Physical Removal of Already Deposited Wax Crystals)**		
	Thermal	Down hole electrical, Heater, Hot oil and Steam injection
	Mechanical	Coiled tube Pigging Jet cutters
	Chemical	Solvent Dispersant
	Hybrid treatment	Mechano-chemical Thermo-chemical

**Table 2:** Classification of drag reducer [55,59,60,61,62,63,64,65,66].

Classification Drag Reduction	Name of Drag Reduction	Ref.
Olefin up to 10 mol %	Low-density polyethylene	[59]
α-Olefins are 1-hexene, 1-octene, 1-decene, and 1-dodecene; cross-linkers are divinylbenzene or organosiloxanes with pendent vinyl groups.	Copolymer of a linear α-olefin with cross-linkers	[60]
Water-soluble drag reducers for emulsions.	Polyacrylamides Poly(alkylene oxide)	[61,62,63]
Esters with C10 to C18 and ionic monomers; reduce friction in the flow of hydrocarbons by a factor of 5 at concentrations of 25 ppm.	Poly(alkyl methacrylate)s	[64,65]
Styrene also includes tert-butylstyrene (drag reducer for hydrocarbon fluids).	Terpolymer of styrene, alkyl acrylate, and acrylic acid or methacrylic acid	[66]

## Data Availability

Not applicable.
